# Aging Reveals a Role for Nigral Tyrosine Hydroxylase ser31 Phosphorylation in Locomotor Activity Generation

**DOI:** 10.1371/journal.pone.0008466

**Published:** 2009-12-23

**Authors:** Michael F. Salvatore, Brandon S. Pruett, Sandy L. Spann, Charles Dempsey

**Affiliations:** Department of Pharmacology, Toxicology, and Neuroscience, Louisiana State University Health Sciences Center, Shreveport, Louisiana, United States of America; Sapienza University of Rome, Italy

## Abstract

**Background:**

Tyrosine hydroxylase (TH) regulates dopamine (DA) bioavailability. Its product, L-DOPA, is an established treatment for Parkinson's disease (PD), suggesting that TH regulation influences locomotion. Site-specific phosphorylation of TH at ser31 and ser40 regulates activity. No direct evidence shows that ser40 phosphorylation is the dominating mechanism of regulating TH activity *in vivo*, and physiologically-relevant stimuli increase L-DOPA biosynthesis independent of ser40 phosphorylation. Significant loss of locomotor activity occurs in aging as in PD, despite less loss of striatal DA or TH in aging compared to the loss associated with symptomatic PD. However, in the substantia nigra (SN), there is equivalent loss of DA or TH in aging and at the onset of PD symptoms. Growth factors increase locomotor activity in both PD and aging models and increase DA bioavailability and ser31 TH phosphorylation in SN, suggesting that ser31 TH phosphorylation status in the SN, not striatum, regulates DA bioavailability necessary for locomotor activity.

**Methodology and Principal Findings:**

We longitudinally characterized locomotor activity in young and older Brown-Norway Fischer 344 F_1_ hybrid rats (18 months apart in age) at two time periods, eight months apart. The aged group served as an intact and pharmacologically-naïve source of deficient locomotor activity. Following locomotor testing, we analyzed DA tissue content, TH protein, and TH phosphorylation in striatum, SN, nucleus accumbens, and VTA. Levels of TH protein combined with ser31 phosphorylation alone reflected inherent differences in DA levels among the four regions. Measures strictly pertaining to locomotor activity initiation significantly correlated to DA content only in the SN. Nigral TH protein and ser31 phosphorylation together significantly correlated to test subject's maximum movement number, horizontal activity, and duration.

**Conclusions/Significance:**

Together, these results show ser31 TH phosphorylation regulates DA bioavailability in intact neuropil, its status in the SN may regulate locomotor activity generation, and it may represent an accurate target for treating locomotor deficiency. They also show that neurotransmitter regulation in cell body regions can mediate behavioral outcomes and that ser31 TH phosphorylation plays a role in behaviors dependent upon catecholamines, such as dopamine.

## Introduction

We face an increased probability of reduced mobility (bradykinesia) with advancing age, with a 50% risk of this Parkinsonian-like symptom by age 85 [1], [2]. The neurobiological basis of this aging-related impairment to locomotion is crucial to resolve, particularly due to the imminent increase of the elderly population. Aging-related Parkinsonism presents symptoms similar to Parkinson's disease (PD) and, like PD, they are progressive, impair performance of essential locomotor functions, and increase risk of cognitive impairment, dementia, and death [2]–[5]. Major dopamine (DA) loss of 70–80% in the striatum is thought to be required for major symptom presentation in PD [6]–[10]. However, in aged humans and animal models alike, striatal DA loss does not reach symptomatic levels seen in PD, ranging instead from 0 to 50% [11]–[15]. Throughout the lifespan there is also little if any aging-related loss of striatal tyrosine hydroxylase ((TH), the rate-limiting enzyme for DA biosynthesis) [15]–[20], which is in stark contrast to the 80% loss seen in symptomatic PD [6], [8]. Thus, it is curious why aging-related Parkinsonism occurs if DA and TH loss are not equivalent to the symptomatic levels seen in PD. If TH loss does not occur in striatum during aging, it is possible that either decreased striatal TH activity may contribute to Parkinsonism, or decreased TH protein or activity in another DA region is the source of Parkinsonism.

The activity of TH is regulated by site-specific phosphorylation, but of the three physiologically-regulated sites in the CNS (ser19, ser31, and ser40) [21], the site most critical for regulating DA biosynthesis in intact neuropil is unknown. Phosphorylation of TH at ser19 does not directly influence TH activity [22], but increased phosphorylation of ser40 can increase TH activity [23]–[26] and is associated with increased DA turnover in neurodegenerative or other CNS disorders [27], [28]. However, a certain threshold of phosphorylation may be required to affect DA biosynthesis [25] and ser40 phosphorylation is not required for stimulation of L-DOPA biosynthesis by depolarizing stimuli [29]. However, increases in ser31 phosphorylation alone increase L-DOPA biosynthesis [25]. Understanding the extent to which ser31 or ser40 phosphorylation modulate DA bioavailability in intact neuropil is necessary to define the molecular basis for DA-dependent behaviors, such as locomotion.

While loss of TH activity may explain variable DA loss in the aged striatum, it is also possible that aging reduces TH protein or activity, and therefore DA bioavailability, in other regions affecting locomotor activity. For example DA signaling in the nucleus accumbens (NAc) affects goal-related locomotor activity [30] as it does in striatum [31]. However, stimulant-induced increases in DA signaling in NAc are similar between aged and young subjects and thus do not explain reduced stimulant-induced locomotion in aged subjects [32]. A potential explaination is that an intact nigrostriatal pathway may be necessary for stimulant enhancement of locomotor activity [33]. In this pathway, dopamine is released not only in striatum, but also in the substantia nigra (SN) [34], [35]. Although striatal DA tissue content is ∼10-fold greater than in SN [12], [13], DA in SN has a longer synaptic life than in striatum [36], [37]. Notably, unlike striatum, aging reduces TH protein in SN and this correlates with motor impairment [38]. Thus TH regulation of DA in the SN may affect certain aspects of locomotor activity generation. In fact, decreasing nigral DA release capacity [39] or blocking its post-synaptic actions [40], [41] reduces locomotor activity. Bradykinesia is a common symptom of PD and aging. Similar deficiencies in nigral DA signaling as well as reduced TH protein reported with the onset of PD symptoms [10], [42] and in aging [18], [38], [43] also point to the possibility that nigral DA bioavailability regulates locomotor activity generation. Aging also reduces nigral TH activity [44], and DA loss ranges from 36%–70% in human [45], non-human primates [17], and rat [13]. Thus, deficits in TH protein and phosphorylation at the key TH activity-modulating site in SN could be the common source of bradykinesia in PD and in aging.

Studies with growth factors indicate that nigral TH activity may regulate locomotor activity. GDNF increases locomotor activity in both aging or PD models in conjunction with increased nigral DA tissue content, notably without affecting striatal DA tissue content [46]–[49]. In aged rats, GDNF produces a large increase in ser31 phosphorylation in the SN [50], suggesting that the action of GDNF is to reverse possible aging-related reductions in nigral ser31 phosphorylation, thereby increasing nigral DA and locomotor activity as reported. Furthermore, unilateral striatal GDNF administration increases TH phosphorylation bilaterally only in the SN and not striatum [51]. This may also occur in human, as bilateral motor improvement is reported following unilateral GDNF delivery in PD patients [52]. Taken together, we hypothesized that ser31 phosphorylation of TH in SN may regulate DA bioavailability necessary for particular aspects of locomotor activity. To test this hypothesis we profiled DA tissue content and TH protein and phosphorylation in the nigrostriatal and mesoaccumbens pathways in young and aged rats following longitudinal characterization of inherent locomotor activity. This design, while correlational, provided an intact and pharmacologically-naïve source of potential endogenous differences in DA bioavailability in DA neuropil in two groups significantly different in locomotor activity to address which TH phosphorylation site normally regulates DA bioavailability and which DA region influences specific aspects of inherent locomotor activity.

## Methods

### 2.1 Test Subject Selection and Handling

Young and old rats were chosen for this study because differences in locomotor activity are derived from intact and pharmacologically-naïve neuropil. Two age groups of Brown-Norway Fisher 344 F_1_ hybrid (BNF) rats arrived at the medical center at one and 19 months of age and given food and water *ad libitum* for three months prior to the start of any locomotor assessment. All locomotor testing was done between the hours of 0900 and 1700. All animals used in this study were drug naïve and other than being tested for locomotor activity 17 times for one hour each session, remained in their home cage, singly housed. The BNF strain was selected for this study because aging-related changes in striatal and midbrain DA tissue content are comparable to that in the primates and exhibits reduced locomotion in aging ([13], [53], and as discussed in main text), making it arguably the best rat model to address the relative role of the striatal and nigral DA in locomotor activity. Outbred Sprague-Dawley rats also show DA loss in the midbrain like the BNF strain [54] but may have increased variance in a variety of measures. The inbred Fischer 344, a commonly used strain in aging studies, does not show any significant tissue loss of DA in striatum or midbrain, contrary to findings in human and non-human primates (although release of DA is compromised 50% [12]). In general, F_1_ hybrids have less variability in a large number of physiological and behavioral measures, including locomotor functions [55]. This reduction in variance in F_1_ hybrids requires fewer animals to attain significance [56].

### 2.2 Assessment of Locomotor Activity

Open-field locomotor activity was determined a total of 17 times in two specific time points, the young being tested at 4–5 months and again eight months later at 11–12 months, and the older group at 22–23 months of age and again eight months later at 29–30 months of age. [Neurochemical measures were made only at the 12 and 30 month ages]. Each rat was tested by pairing with the same different age counterpart in separate locomotor test chambers (10 trials in the first period of life and 7 trials in the second period of life eight months later). Spontaneous locomotor activity was assessed in 1 hour duration trials using automated activity chambers (VersaMax Animal Activity Monitoring System, Columbus, OH), which consisted of a 41×41×31 cm^3^ plexiglass box with a grid of infrared beams mounted horizontally every 2.5 cm and vertically every 4.5 cm. The beams are generated from monitors connected to a VersaMax analyzer which transmits the number of beam breaks (locomotor activity) to a dedicated computer with VersaMax software to collect and analyze the locomotor measures. Activity data was recorded in four consecutive 15 min samples and the sum total for movement number, total distance (cm), horizontal activity (unitless measure of all horizontal activity), time spent moving (sec) were recorded. Movement speed was calculated by dividing total distance by time spent moving (cm/sec). The typical life-expectancy of the BNF rat is approximately 32 months and thus, the locomotor activities in about one-fourth to one-third of the average life span of the rat were determined for each age group. Combining both operationally-matched age groups, the locomotor activity patterns of virtually the entire adult life span was determined.

To assess DA tissue content and TH protein and phosphorylation, within 5–10 min following the last (17^th^) locomotor test session, the rats were killed in alternating order of young or old, and dorsal striatum, substantia nigra, nucleus accumbens, and VTA were dissected from 1 mm coronal slices as previously described [57] and stored on dry ice and −70°C until processed for DA analysis by HPLC, protein levels, and phosphorylation analysis by western blotting.

### 2.3 Neurochemistry: Dopamine Analysis

The analysis of DA tissue content from striatum, SN, NAc, and VTA allowed for two major objectives to be addressed; 1) to determine how TH phosphorylation controls DA biosynthesis capacity in the CNS, and 2) to correlate to measures of locomotor activity with DA tissue content in these four regions. Given the well-established DA hypothesis for locomotion, this experimental design makes it possible to determine whether DA in one or more of these regions influenced locomotion. Our operationally-matched assessment of DA tissue content and TH measures from all four brain regions from each test subject is a novel state-of-the-art approach to equate the DA tissue content resulting from a respective TH protein and TH phosphorylation profile and to determine their contributions to locomotor activity. This addition of locomotor measures to the neurochemical profile is the first known study of its kind, which adds a behavioral component in assessing DA tissue content with TH protein and TH phosphorylation [27].

Tissues were sonicated in ice-cold 0.1 M HClO_4_-EDTA buffer. Aliquots from these protein-precipitated supernatants were analyzed for content of DA by HPLC [58]. Determination of DA is calculated based upon percent recovery of the internal standard (N-methyl-dopamine (NMDA), 20 ng/mL) in each sample, the peak height ratios obtained for both DA and NMDA in a standard and in the sample, and the fraction of the aliquot analyzed by HPLC representing the total volume of the sonicated tissue. The acid buffer treatment for HPLC analysis precipitates the protein content inherent in the sample, and this protein pellet was sonicated in 1% SDS with 5 mM Tris buffer (pH 8.3) and 1 mM EDTA to determine total protein, TH protein, and site-specific TH phosphorylation.

We normalized total DA to protein, as opposed to the wet weight of tissue. This was done to control for variances that can arise from contributions of non-protein material. However, this normalization also requires precision dissections for each test subject along the rostral-caudal axis, as the inadvertent additions of non-DA cells in the dissection would increase protein content not related to DA neuropil. For this reason, we present the DA measures as per total DA harvested and as total DA normalized to mg protein. These results were similar (Table 1) and had significant correlation with each other (data not shown). Furthermore, our additional normalization of total DA to total TH harvested in the sample is the strictest normalization to DA neuropil and gives the best insight into endogenous TH activity.

**Table 1:** *p*-values of locomotor functions versus DA tissue content.

		*nigrostriatal*			
		Striatum		SN	
Parameter	variable	DA	DA/prot	DA	DA/prot
*Movement number*	Lifetime avg	0.66	0.48	**0.008**	**0.028**
	Session 2	0.83	0.59	**0.005**	**0.042**
	Highest lifetime	0.94	0.43	**0.004**	**0.011**
	Lowest lifetime	0.51	0.35	**0.007**	**0.010**
	Last trial	0.42	0.28	**0.011**	**0.012**
*Total distance*	Lifetime avg	0.35	0.32	**0.026**	**0.012**
	Session 2	0.36	0.68	**0.035**	**0.024**
	Highest lifetime	0.97	0.99	**0.009**	**0.017**
	Lowest lifetime	0.24	0.41	**0.031**	**0.042**
	Last trial	0.38	0.62	0.15	**0.034**
*Horizontal activity*	Lifetime avg	0.58	0.56	**0.004**	**0.016**
	Session 2	0.78	0.90	**0.003**	**0.038**
	Highest lifetime	0.94	0.74	**0.002**	**0.016**
	Lowest lifetime	0.59	0.42	0.07	0.15
	Last trial	0.52	0.46	**0.007**	**0.005**
*Time spent moving*	Lifetime avg	0.42	0.41	**0.013**	**0.010**
	Session 2	0.93	0.75	**0.011**	**0.038**
	Highest lifetime	0.94	0.70	**0.002**	**0.010**
	Lowest lifetime	0.27	0.31	**0.019**	**0.029**
	Last trial	0.42	0.51	**0.026**	**0.012**
*Movement speed*	Lifetime avg	0.06	0.95	0.44	**0.047**
	Session 2	0.055	0.73	0.60	0.065
	Highest lifetime	0.76	0.25	0.54	0.58
	Lowest lifetime	0.56	0.33	0.26	0.19
	Last trial	0.67	0.12	0.051	0.46

Correlation statistics (*p*-values derived from Spearman correlation) of locomotor activity measures to respective DA tissue content (total ng or total ng per protein) in striatum and SN analyzed in 7 paired 12- and 30-month old rats. Activity measures are further subdivided into the *lifetime* average (mean of 17 total locomotor trials conducted at 4 and 12 months for the young group and 22 and 30 months for the old group), *session 2* (mean of seven locomotor trials that were conducted at 12 and 30 months), *highest and lowest lifetime* (highest ever, and lowest ever, locomotor activity measures of the 17 sessions), and *last trial* (the final locomotor activity measure of the 17, recorded immediately prior to tissue dissection). Significant correlations are in bold.

### 2.4 Neurochemistry: Determination of TH Protein and Phosphorylation

Protein levels were determined by BCA method and samples were prepared in reducing (dithiothreitol used as reducing agent) sample buffer containing SDS and subjected to SDS gel electrophoresis on 10% gels, and transferred to nitrocellulose. Protein loads were verified by Ponceau S stain and blots were preincubated with polyvinylpyrrolidone-based Tris buffer for at least 2 hr prior to antibody exposure to reduce non-specific binding. For each blot immunolabeling experiment, after treatment with primary and secondary antibody (for signal enhancement), the detection method used I^125^-protein A (high-specific activity). The blots were exposed to Kodak film which reveals immunoreactive areas on the blot. These areas were excised and counted for gamma radioactivity. To quantify total TH, in house standards (calibrated PC12 cell extract) are used to interpolate sample immunoreactivity to anti-TH versus a standard curve of known quantities of TH and the value is expressed as ng TH per ug total protein loaded [25], [27], [50], [51], [57].

Protein phosphorylation is quantified by western blot using affinity-purified primary antibodies specific to each phosphorylation site. We used our own affinity-purified ser31 phosphorylation primary (21^st^ Century Biochemicals), and ser19 and ser40 antibodies were a gift from Dr. John W. Haycock. The ser31 primary antibody was validated for phosphorylation-state specificity (Fig. S1, Text S1). Calibrated phosphorylation site-specific in house standards are used to quantify phosphorylation levels and are normalized to the TH content to determine the phosphorylation stoichiometry, as previously reported [25], [27], [50], [51], [57]. Assays for TH protein and its site-specific phosphorylation are conducted within the dynamic working range of each antibody.

### 2.5 Statistics

#### Analysis of locomotor profiles

Locomotor activity was analyzed in five parameters. Four parameters (movement number, total distance, horizontal activity, and time spent moving) were recorded with software as previously described and movement speed was calculated as total distance divided by time spent moving. Aging related comparisons were analyzed by ANOVA followed by Bonferroni's multiple comparison test between the age groups and also a Student's t-test was used for within age-group comparisons (4 vs. 12 months, 22 vs. 30 months). Correlations between dependent measures used the Spearman correlation coefficient, including correlations of locomotor activity with DA measures, as shown in table 1. The five parameters of locomotor activity for each test subject were also analyzed in five ways (lifetime average, average of second session of seven trials, highest lifetime trial result, lowest lifetime trial result, and the last (17^th^) trial.

#### Analysis of neurochemical profiles

Dopamine tissue content was analyzed three ways; 1) total DA recovered per region, 2) total DA recovered per total protein, 3) total DA recovered per total TH. The respective tissue content of DA in the four regions are considered treatments for the purpose of determining the influence of TH protein and site-specific phosphorylation upon DA tissue content. The role of site-specific phosphorylation upon DA tissue content was determined by normalizing inherent DA to TH protein and comparing differences in site-specific phosphorylation to the differences in DA per TH protein in each region (ANOVA followed by Bonferroni's multiple comparison test). We also compared total DA harvested in each region with total TH protein harvested to show how TH protein alone contributes but does not fully account for the regional differences in DA.

## Results

### 3.1 Molecular Origin of DA Bioavailability

**Figure 1:**
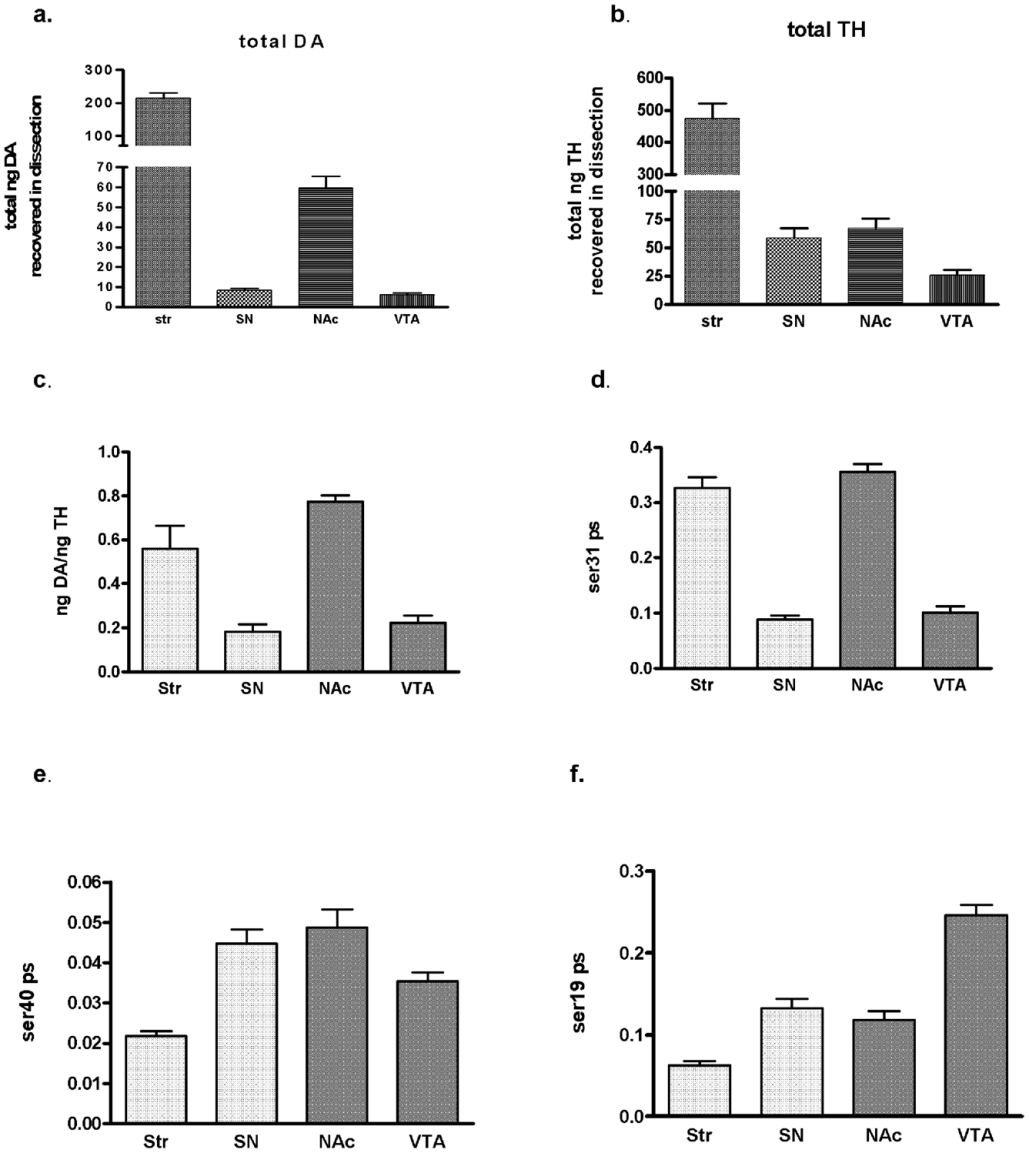
Dopamine, tyrosine hydroxylase protein, and phosphorylation stoichiometry (ps) comparisons in nigrostriatal and mesoaccumbens pathways. Analyses of striatum (str), substantia nigra (SN), nucleus accumbens (NAc), and ventral tegmental area (VTA) from all test subjects (*n* = 14, seven pairs of 12 and 30 month old rats from locomotor study). **a. total DA recovery** The mean relative total recovery of DA (in ng) was Str = 214±16; SN = 8.4±0.8; NAc =  59.6±5.8; VTA = 6.0±1.0. **b. total TH protein recovery** The mean TH recovery (in ng) was Str = 473±49; SN = 59±9; NAc = 68±9; VTA = 26±5. **c. TH-Normalized DA tissue content** Str 0.56±0.11, SN 0.18±0.03, NAc 0.77±0.03, VTA 0.22±0.03. There was a significant difference in DA recovered per TH among the four regions (ANOVA (p<0.0001) and between Str and SN (*p*<0.001), Str and VTA (*p*<0.01), SN and NAc (*p*<0.001), and VTA and NAc (*p*<0.001). **d. ser31 phosphorylation stoichiometry (PS)** Str 0.33±0.02, SN 0.09±0.01, NAc 0.36±0.01, VTA 0.10±0.01. There was a significant difference in ser31 phosphorylation among the four regions (ANOVA (p<0.0001) and between Str and SN (*p*<0.001), Str and VTA (*p*<0.001), SN and NAc (*p*<0.001), and VTA and NAc (*p*<0.001). **e. ser40 PS** Str 0.022±0.001, SN 0.045±0.004, NAc 0.049±0.004, VTA 0.035±0.002. There was a significant difference in ser40 phosphorylation among the four regions (ANOVA (p<0.0001) and between Str and SN (*p*<0.001), Str and NAc (*p*<0.001), Str and VTA (*p*<0.05), and NAc and VTA (*p*<0.05). **f. ser19 PS** Str 0.06±0.01, SN 0.13±0.01, NAc 0.12±0.01, VTA 0.25±0.01. There was a significant difference in ser19 phosphorylation among the four regions (ANOVA (p<0.0001) and between Str and SN (*p*<0.001), Str and NAc (*p*<0.01), Str and VTA (*p*<0.001), SN and VTA (*p*<0.001) and NAc and VTA (*p*<0.001).

Although phosphorylation of TH by ERK at ser31 or PKA at ser40 can increase DA biosynthesis *in situ* [25], [26], which phosphorylation site affects DA bioavailability in intact neuropil is unknown. As would be predicted, though not yet shown *in vivo*, total TH protein content in the SN correlated to total DA recovered in each test subject (*p*<0.05, Fig. S2) and significant correlations were also seen in the other three regions (data not shown). However, relative differences in DA tissue content among the four regions (Fig. 1a) were not matched by TH content in these tissues (Fig. 1b) [note that actual values are indicated in figure legend to support this finding], signifying that site-specific phosphorylation also contributes to regulating inherent DA tissue content in the CNS. Normalizing DA tissue content to total TH recovered in each region removes the influence of total TH on DA content (Fig. 1c). Therefore, the phosphorylation site regulating TH activity *in vivo* in intact neuropil would be predicted to vary across all four regions in a pattern similar to the differences in TH-normalized DA content. The differences in ser31 phosphorylation stoichiometry (Fig. 1d) reflected these differences in TH-normalized DA content, whereas ser40 (Fig. 1e) and ser19 (Fig. 1f) phosphorylation stoichiometry did not reflect these differences.

Total TH protein also affected total DA content in conjunction with ser31 phosphorylation. Total DA recovered from the four tissues was greatest in the striatum, followed by the nucleus accumbens ((NAc) ∼3.5-fold less than striatum), and comparable amounts in the SN and VTA (Fig. 1a). Recovery of DA in the NAc was nearly seven-fold greater than in SN (Fig. 1a) despite relatively equal recovery of TH protein between the NAc and SN (68 and 59 ng total TH, respectively (Fig. 1b)), and this difference was explained by ∼four-fold greater ser31 phosphorylation stoichiometry (Fig. 1d) in the NAc compared to SN (*p*<0.001, Fig. 1d), whereas ser19 or ser40 phosphorylation stoichiometry was not significantly different between the two regions (Figs. 1e, 1f). However, total DA recovery in striatum was 3.5-fold greater than in the NAc (Fig. 1a), in spite of similar levels of ser31 phosphorylation stoichiometry (Fig. 1d). Between these regions it appears that the 3- to 4-fold greater level of TH protein in striatum compared to NAc (Fig. 1b) influenced their inherent differences in DA.

**Figure 2:**
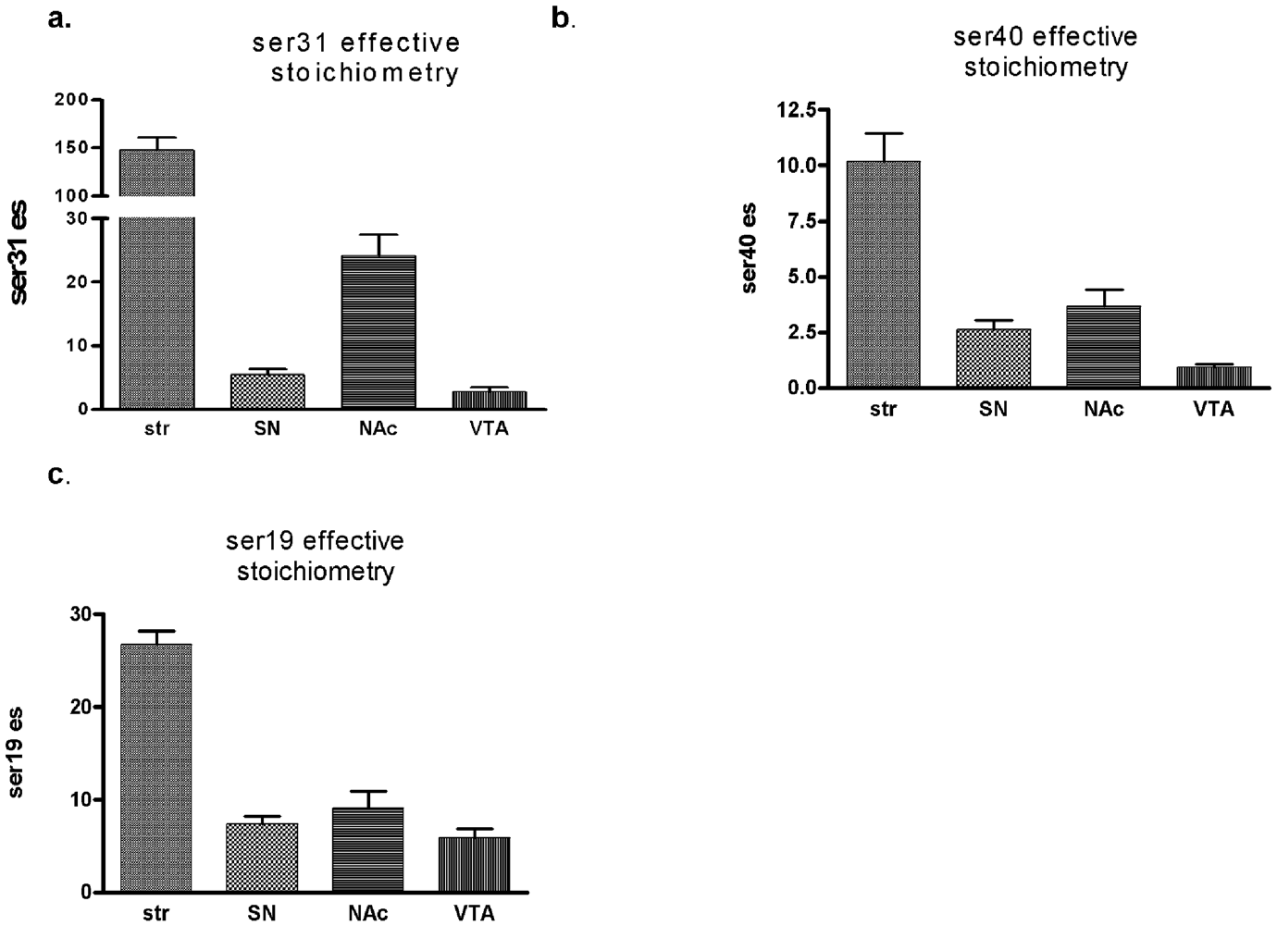
Tyrosine hydroxylase effective stoichiometry in nigrostriatal and mesoaccumbens pathways. Values are obtained by multiplying total inherent TH protein content by phosphorylation stoichiometry in each sample. **a.** **Ser31 es**. Stoichiometry determined by anti-phospho ser31 TH against calibrated standards for ser31 and divided by total TH protein. **b. Ser40 es c. ser19 es** Values were calculated based upon each subject's ps value and multiplied by the mean total TH. Region-to-region difference for ser31 es was similar to the difference in total DA (as shown in **Fig. 1a**) recovered from the longitudinally-characterized inherent locomotor activity patterns for the test subjects.

These data show that both TH protein levels and phosphorylation at ser31 in intact CNS tissue affect DA tissue content. Thus, multiplying the total TH protein content by TH phosphorylation stoichiometry (effective stoichiometry) at the key regulatory phosphorylation site should represent inherent DA biosynthesis capabilities in DA neuropil. The effective stoichiometry at ser31 should, therefore, reflect the differences in DA content across each region. Indeed, the effective stoichiometry for ser31 (Fig. 2a), unlike ser40 or ser19 (Fig. 2b, 2c), had similar region-to-region variance as compared to differences in total DA content (Fig. 1a). Notably, this is apparent in comparing striatum to SN, as there was a 25-fold greater level of DA in striatum (Fig. 1a) and a nearly identical 24-fold difference in effective stoichiometry at ser31 (eight-fold greater TH protein (Fig. 1b) times three-fold greater ser31 phosphorylation stoichiometry (Fig. 1d)). The effective stoichiometry at ser40 (Fig. 2b) was only four-fold different between striatum and SN and was not significantly different between SN and nucleus accumbens and as such did not reflect the differences in DA tissue content recovered (Fig. 1a). These observations clearly show that ser31 phosphorylation, in conjunction with TH protein content, regulates DA bioavailability in the intact nigrostriatal and mesoaccumbens dopaminergic neuropil. In terms of behavioral significance, these findings suggest that ser31 phosphorylation status may drive DA-dependent behaviors from neuroanatomical loci associated with such behaviors.

### 3.2 Locomotor Profiling and Regional Dopamine Bioavailability Correlations

Prior to neurochemical analyses, the test subjects, seven young and seven old BNF rats (groups differing in age by 18 months), were characterized for open-field locomotor activity, independent of exogenous influences such as systemically- or CNS-delivered drugs, in a longitudinal study. There were ten trials conducted when the young group was 4 months old and the old group was 22 months old, and another seven trials conducted when the young group was 12 months old and the old group was 30 months old. The paucity of initiated movement is a signature behavioral symptom of bradykinesia, and as such, movement number, horizontal activity, and time spent moving are three locomotor activity measures arguably the closest to quantifying bradykinesia. Notably, movement number had significant correlation with all other measures of locomotor activity at all ages of testing, with the exception of movement speed, (Fig. S3). The repetitive nature of the locomotor activity measures did not produce a significant decrease in locomotor activity in the majority of the test subjects (Table S1) in either testing session, so habituation was not a confounding effect on mean locomotor results.

**Figure 3:**
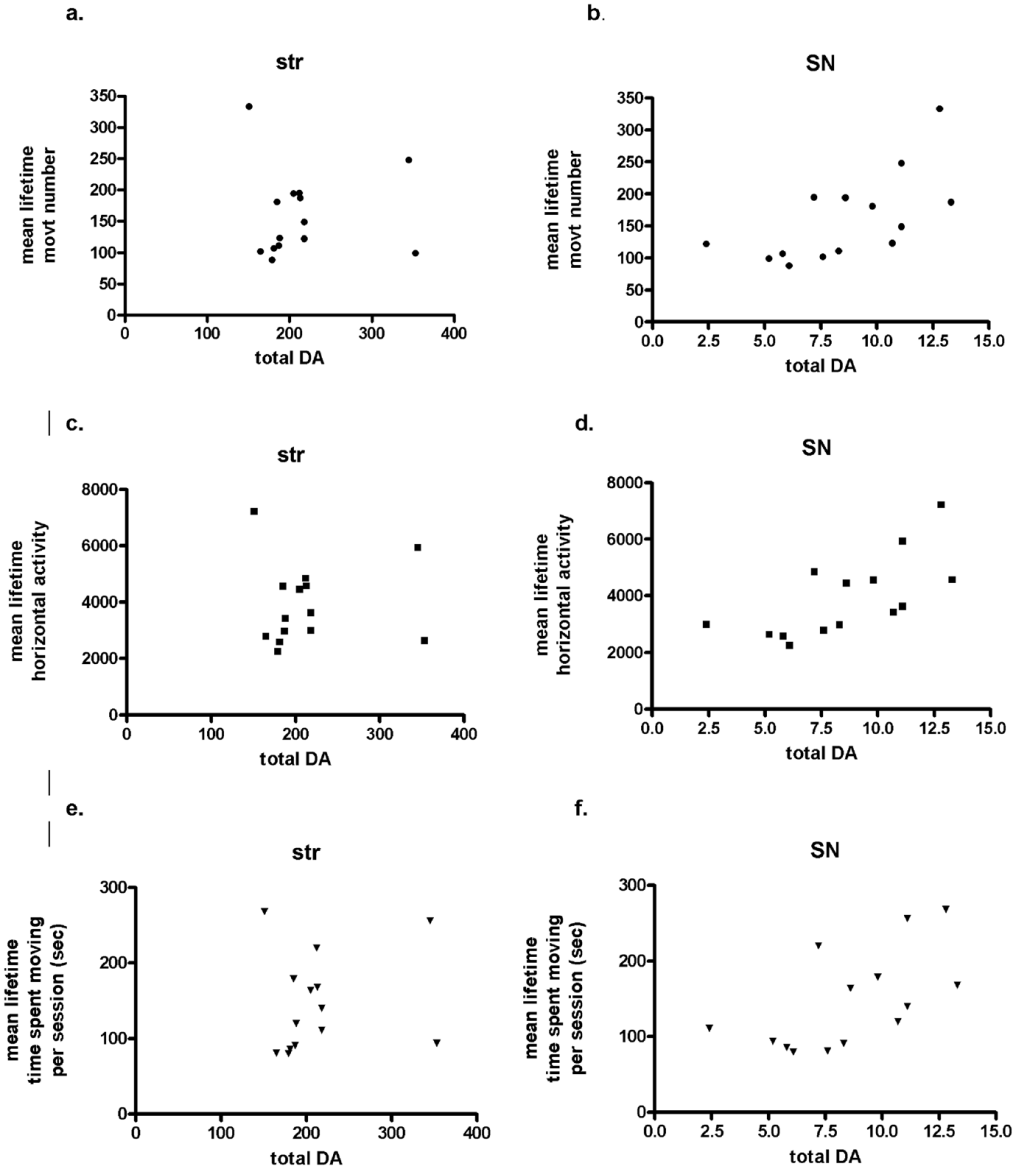
Dopamine bioavailability and correlation to locomotor activity generation. Total DA in striatum ((str) a, c, e) versus substantia nigra ((SN) b, d, f) and correlation to mean lifetime locomotor activity measures in all 14 subjects, ages 12 and 30 months (*n* = 7 each age group). The following are the Spearman correlation values and *p*-values; **Movement number, a. str**, *p* = 0.65, r = 0.13 **b. SN**, **p = 0.008, r = 0.68, **Horizontal activity, c. str**, *p* = 0.58, r = 0.16 **d. SN**, **p = 0.008, r = 0.72; **Time spent moving, e. str**, *p* = 0.42, r = 0.24 **e. SN**, *p = 0.01, r = 0.64. Additional statistical correlations are presented in Table 1.

Because there was only one opportunity to assess DA tissue content along with TH protein and TH phosphorylation, we compared the locomotor activity results in five time-related measures; *lifetime* average (mean of 17 total locomotor trials conducted at 4 and 12 months for the young group and 22 and 30 months for the old group), *session 2* (mean of seven locomotor trials conducted at 12 and 30 months), *highest and lowest lifetime* (highest ever, and lowest ever, locomotor activity measures of the 17 sessions), and *last trial* (the final locomotor activity measure of the 17, recorded immediately prior to tissue dissection) (Table 1). Total DA tissue content from the SN, but not striatum, NAc, or VTA (NAc and VTA data in Table S2), correlated significantly with four of five locomotor parameters, the notable exception being movement speed (Fig. 3, Table 1), and for each time-related comparison. Striatal DA did not correlate with any locomotor parameter, but there was a noted trend with the lifetime average and session 2 movement speed (Table 1). This lack of correlation may be related to the small insignificant aging-related loss of striatal or accumbal DA.

### 3.3 Relative Individual Locomotor Activities Are Maintained but Decline with Aging

**Figure 4:**
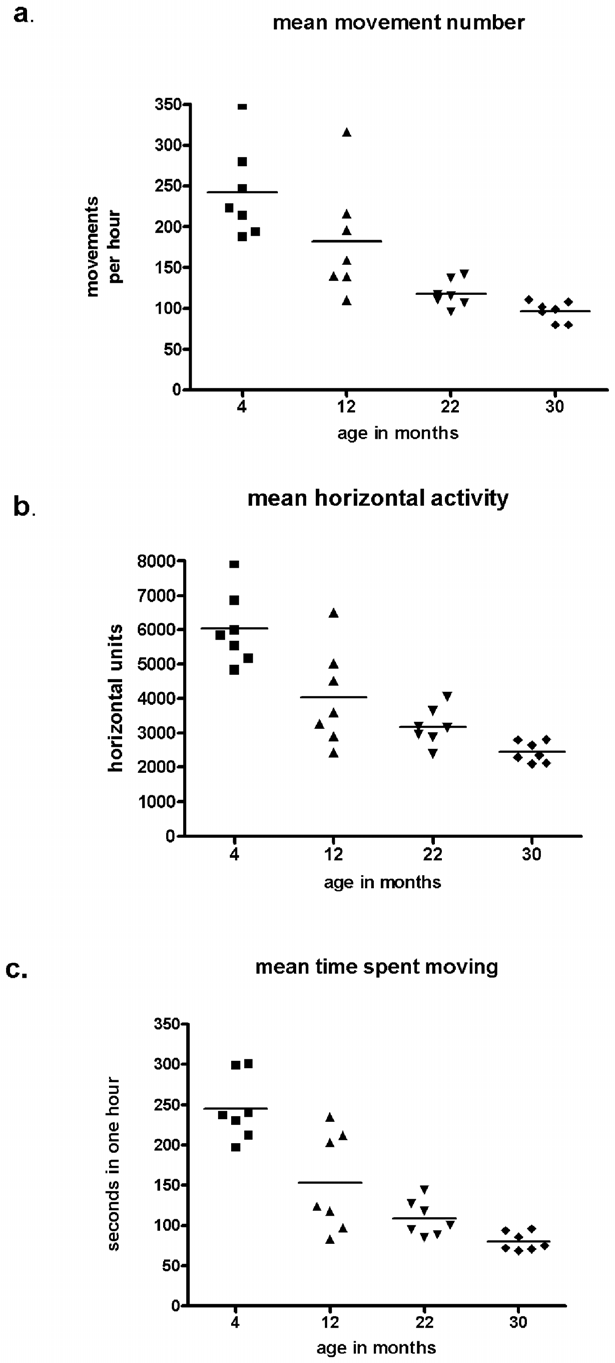
Longitudinal-characterization of locomotor activity during aging. Mean for the age tested represented by horizontal line and the locomotor activities of test subjects represented by points. The young group was tested at 4 and 12 months and the old group tested at 22 and 30 months. **a.** *movement number* (ANOVA, group *p*<0.0001; *post-hoc* 12 vs. 30 mos, *p*<0.05): aging difference within group, 4 vs. 12 mos., *p*<0.05; 22 vs. 30 mos., *p*<0.05 (Student's paired t-test used for all within group comparisons). **b.** *horizontal activity* (ANOVA, group *p*<0.0001; *post-hoc* 12 vs. 30 mos., *p*<0.01): aging difference within group, 4 vs. 12 mos., *p*<0.05; 22 vs. 30 mos., *p*<0.05. **c.** *time spent moving* (ANOVA, group *p*<0.0001; *post-hoc* 12 vs. 30 mos., *p*<0.05): aging difference within group, 4 vs. 12 mos., *p*<0.05; 22 vs. 30 mos., *p*<0.05. **Data not shown**: *total distance* (ANOVA, group *p*<0.0001; *post-hoc* 12 vs. 30 mos, *p*<0.05): age difference within group, 4 vs. 12 mos., *p*<0.05; 22 vs. 30 mos., *p*>0.05, and *movement velocity* (ANOVA, group *p*<0.005; *post-hoc* 12 vs. 30 mos., *p*>0.05): aging difference within group, 4 vs. 12 mos., *p*<0.05; 22 vs. 30 mos., *p*<0.05.

Consistent with the literature, significant aging-related decreases occurred in all locomotor measures with the exception of movement speed. Movement number, horizontal activity, time spent moving, (Fig. 4a–c), and total distance traveled (data not shown) decreased significantly between the age groups (ANOVA, *p*<0.0001). Importantly, there was a significant group difference between the two age groups at 12 and 30 months (*p*<0.05), the ages at which neurochemical profiles were obtained. There was a significant difference at 4 and 22 months as well (*p*<0.001, Bonferroni's multiple comparison test), broaching the possibility of an inherent molecular basis for locomotor activity. The significant group differences in locomotor activity, in addition to the variances seen within groups, gave a range of inherent differences in locomotor activity to address this possibility.

**Figure 5:**
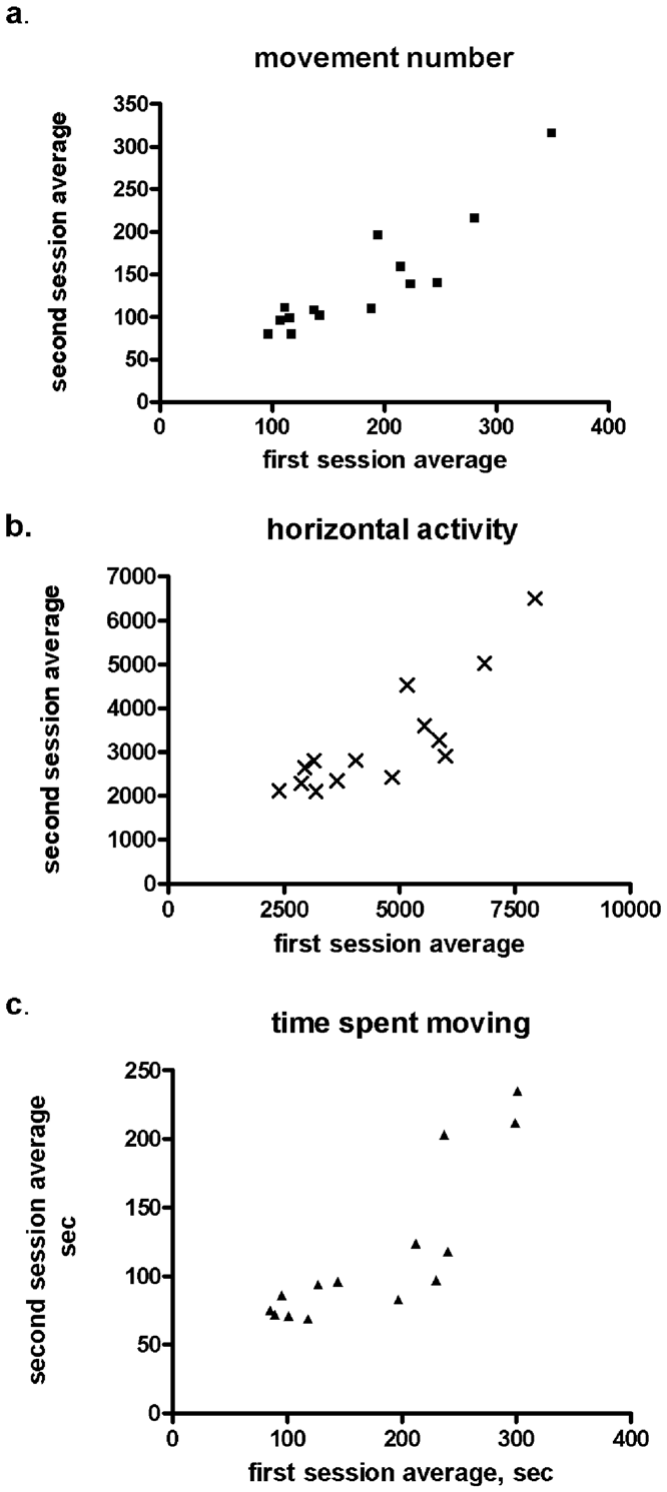
Individual locomotor activities with respect to group throughout the life span. Young adult and old rats were tested at 4 and 22 months old, and again at 12 and 30 months old. There was a significant correlation in locomotor activity between the mean performance for each test subject at the first testing period (ten trial average) and second testing period (seven trial average) of locomotor activity tested eight months later: **a.** movement number, *p*<0.0001, r = 0.865; **b.** horizontal activity (HAC), *p* = 0.0001, r = 0.846; **c.** time spent moving (as seconds per hour), *p*<0.0001, r = 0.868. Data not shown are movement speed (cm/sec), *p* = 0.0001, r = 0.848, and distance traveled (cm/hr), *p*<0.0001, r = 0.881.

While there was an aging-related decline in locomotor activity (Fig. 4), individual test subjects had consistent locomotor activity levels in relation to the other test subjects between the eight months of locomotor trials, suggesting that there is a neurobiological basis for locomotion maintained with respect to the group, though declining, in each individual with advancing age. There was a significant correlation for movement number, horizontal activity, time spent moving (Fig. 5a–c), total distance and movement speed (data not shown) between the first and second test sessions. Thus comparisons of DA and TH measures to inherent lifetime locomotor patterns of test subjects could reveal how TH regulates DA bioavailability and inherent locomotor activity.

### 3.4 Aging, Population Variance, and a Dopaminergic Basis

**Figure 6:**
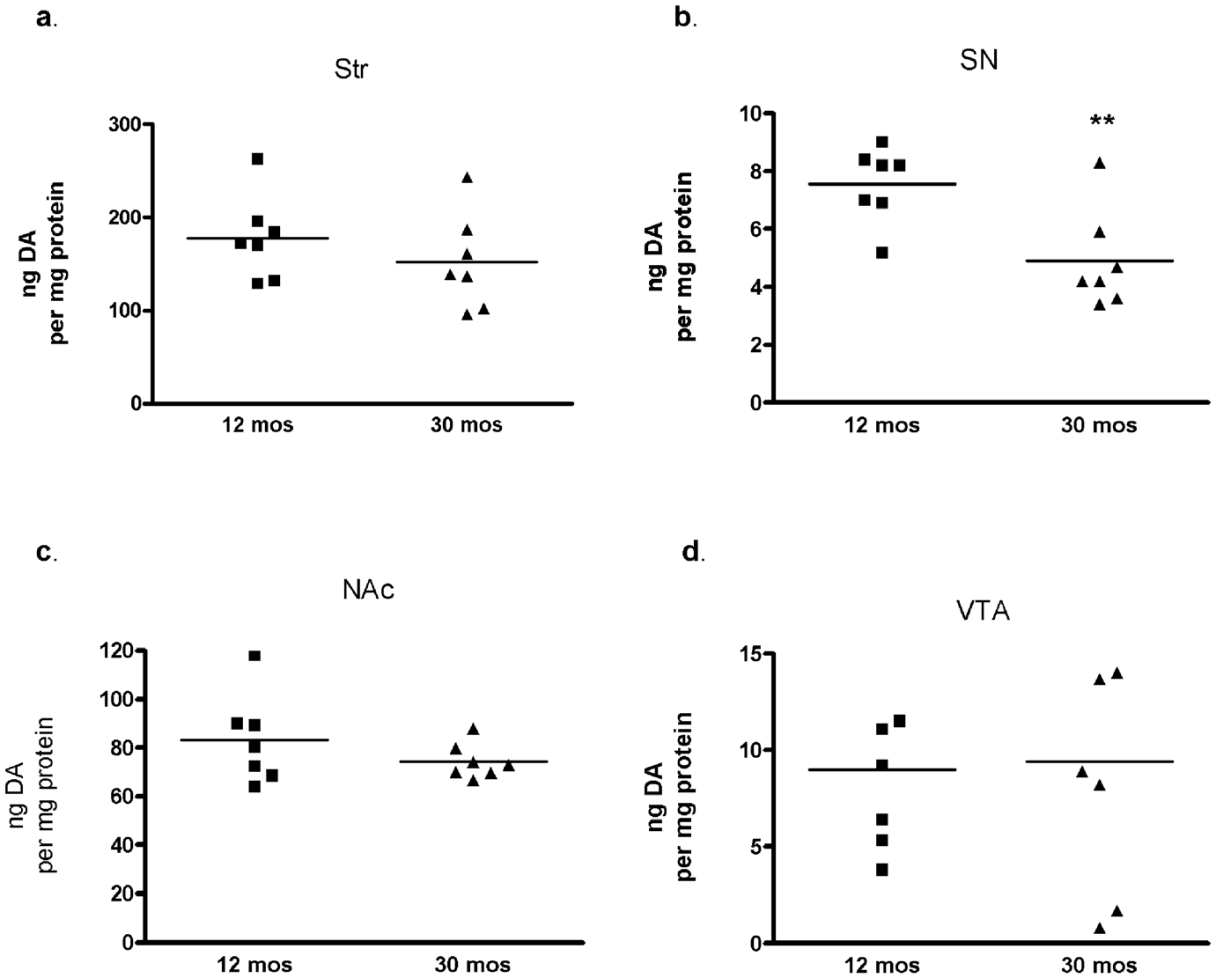
Aging and DA tissue content. Values are ng DA/ mg protein in 12- and 30-month old subjects in the nigrostriatal (a, b) and mesoaccumbens (c, d) dopaminergic pathways. **a.** *striatum*, ns, 12 mo.,178±17, 30 mo., 152±19, **b.** *substantia nigra*, **p<0.01, 12 mo., 7.6±0.5, 30 mo. 4.9±0.6, **c.** *nucleus accumbens*, ns, 12 mo., 83±6.9, 30 mo. 74±2.8, **d.** *ventral tegmental area*, ns, 12 mo., 9.0±1.5, 30 mo., 9.4±2.5. These statistical relationships (Student's t-test, unpaired) also held in the comparison of total DA recovery (not normalized to protein) in each region (Fig S4).

Aged rats have an inherent decrease in locomotor activity and provide intact neuropil to compare where DA bioavailability affects locomotor activity measures. We found that DA levels were significantly lower only in the SN (36% decrease including outlier in 30-month group and 43% decrease without it (Fig. 6)). The levels of DA between the 12 and 30 month old groups trended lower in the striatum (15% decrease) and NAc (11% decrease), but, like those in VTA, were not significantly different. Of note, the nigral DA value in the outlier subject of the older group was at the 12-month mean (8.3 ng/mg protein) and this subject had the highest locomotor activity level of the group. Total DA levels (not normalized to total protein) in SN also decreased significantly in the 30 month group (Fig. S4). Therefore, the significant decrease in overall DA bioavailability occurring in the 30 month group only in the SN implies that ser31 phosphorylation is also decreased in aged SN, given the importance of ser31 phosphorylation in regulating DA bioavailability in intact neuropil (Figs. 1, 2).

### 3.5 The Role of TH in Locomotor Activity

**Figure 7:**
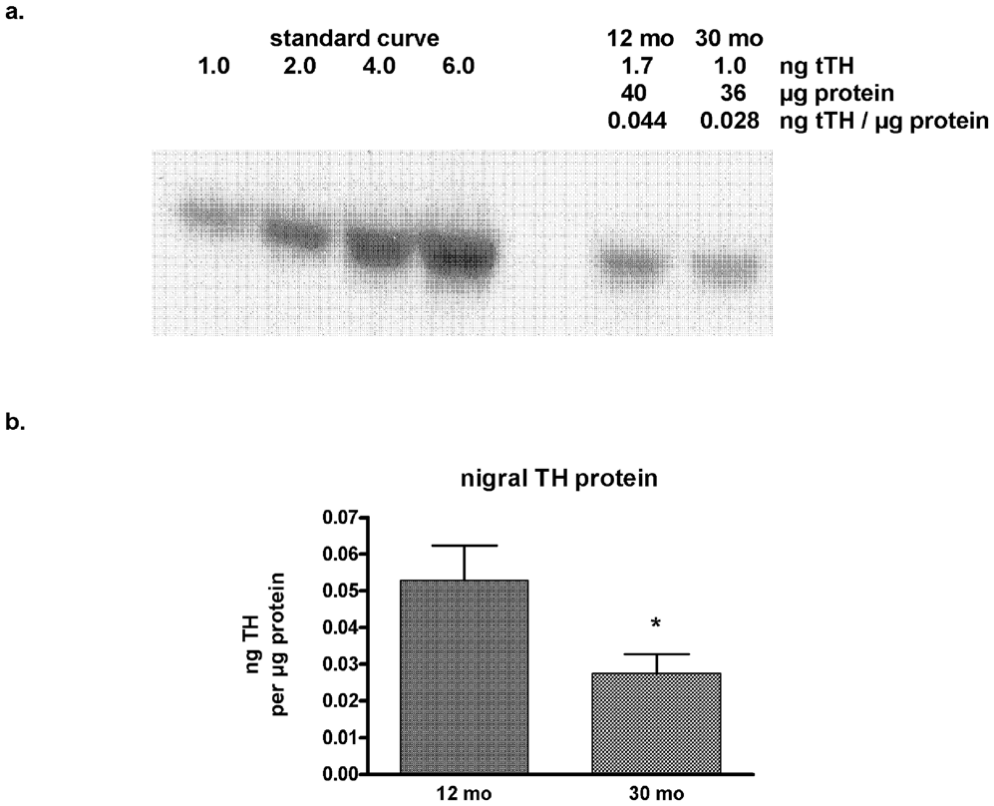
Nigral TH protein loss in aging. **a.** Representative blot showing the ∼40% decrease in total TH (tTH) protein in the SN in a 30-month old BNF rat compared to the tTH content in the 12-month old. All TH protein assays are conducted against a standard curve with quantified TH protein. A specific quantity of total protein is loaded from the sample to determine the ng tTH per µg protein and the quantity from the sample is interpolated from the standard curve. **b.** nigral total TH protein content between the 12- and 30-mo old subjects, 0.053±0.010 and 0.028±0.005 ng per µg protein, respectively. *p*<0.05 (Student's t-test, unpaired).

**Figure 8:**
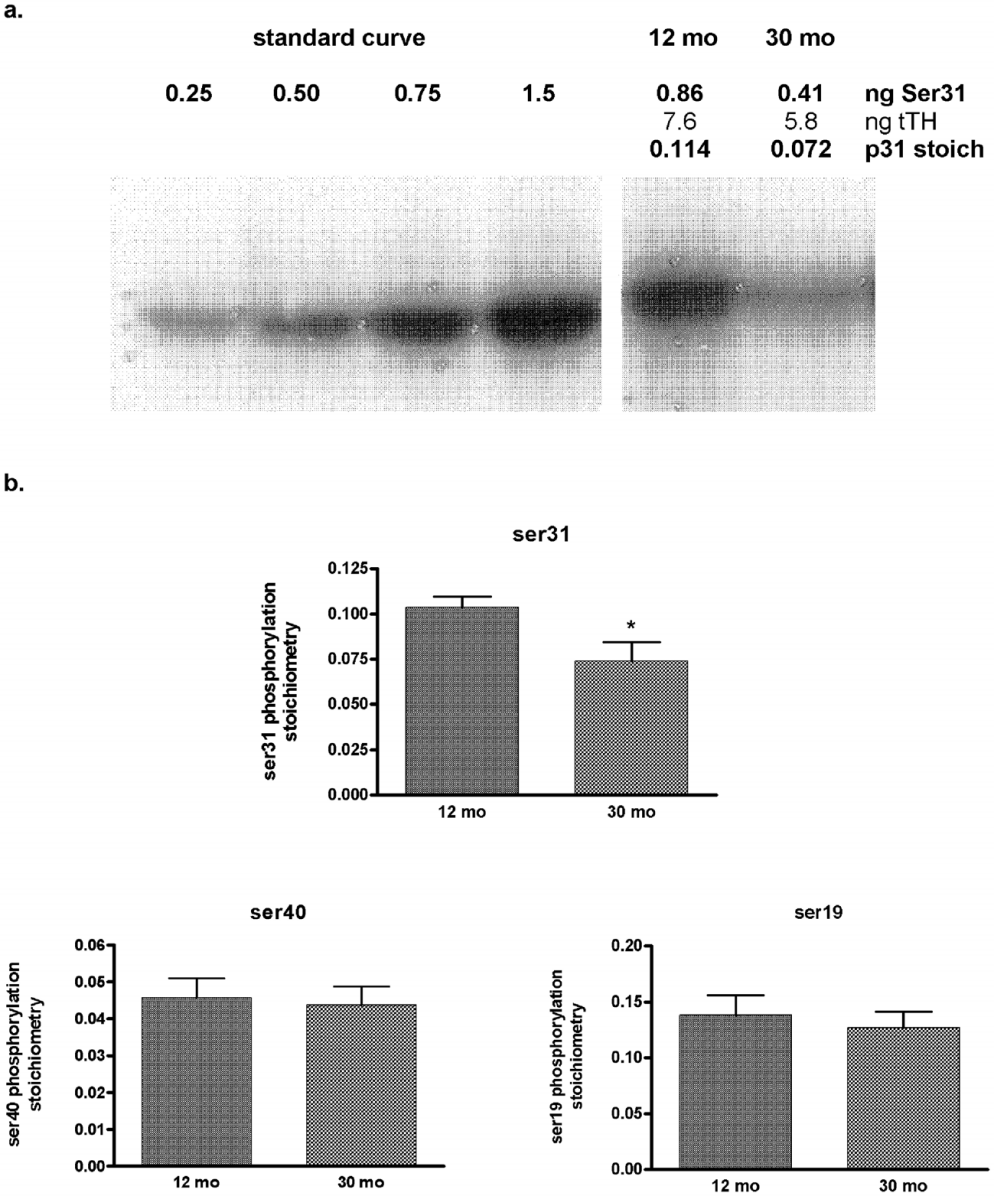
Nigral TH phosphorylation stoichiometry in aging. **a.** Representative blot showing the 30% decrease in TH phosphorylation at ser31 in the 30-month old BNF rat. All TH phosphorylation assays are conducted against a standard curve with quantified site-specific phosphorylation levels so actual quantities of phosphorylated TH at each phosphorylation site are loaded in the standard curve range and the quantity of phosphorylated TH from the sample is interpolated from the standard curve. Because actual quantities of total TH (tTH) vary from sample-to-sample, it is not always possible to load equal amounts of tTH. Total protein load among samples is kept within 25 µg if at all possible to keep any carrier protein effects upon the signal to an absolute minimum. In this case, total protein load differed by 10 µg (80 µg for the 12 mo sample and 90 µg for the 30 mo sample). The standard curve source is from a calibrated PC12 cell extract with a phosphorylation stoichiometry at ser31 at 0.09 ng p31 per ng tTH. **b.** Site-specific phosphorylation of TH in the SN in test subjects. The 30-month group had significantly reduced phosphorylation at ser 31 (*p* = 0.029, unpaired Student's t-test). No significant change was observed at ser40 or ser19.

Nigral TH protein content alone did not significantly correlate to locomotor activity measures (data not shown). This was not particularly surprising given the involvement of phosphorylation at ser31 in regulating bioavailability of DA in the CNS (Figs. 1, 2). There was a significant decrease in TH protein in the SN in the 30 month old group, when the previously mentioned outlier was removed from this group (*p*<0.05) (Fig. 7a, b), consistent with reports as discussed in the introduction. [The total TH protein in this subject accounted for significantly elevated DA levels, as ser31 phosphorylation in this subject was only 14% above the group mean.] No significant age-related loss of TH protein was seen in the other three regions (data not shown). There was also a 30% reduction in ser31 phosphorylation in the SN in the 30-month group (*p*<0.05) (Fig. 8a,b) and, as a result of the lower TH protein and ser31 phosphorylation, there was a 44% reduction in the effective stoichiometry of ser31 (*p*<0.05) (data not shown). This was identical to the actual decrease in DA levels in the SN (Fig. 6b). Other than a <20% reduction in ser19 phosphorylation in VTA and ser31 phosphorylation in the NAc, no other significant aging-related changes in phosphorylation were observed in the four brain regions (data not shown).

**Figure 9:**
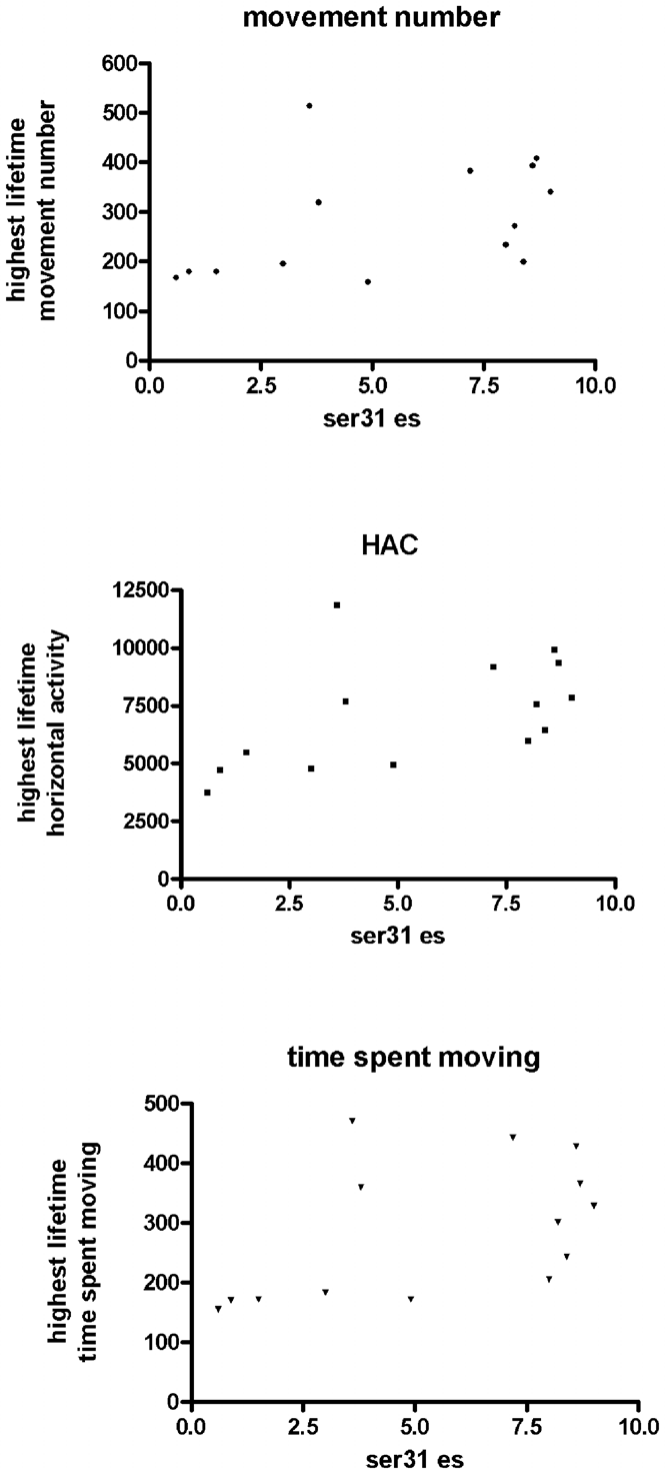
Effective stoichiometry at ser31 and locomotor activity. Effective stoichiometry of ser31 (the product of ser31 phosphorylation stoichiometry and TH protein quantity) correlates with the highest ever recorded locomotor activity measures (movement number, horizontal activity, and time spent moving) of the 17 trials for each test subject. Spearman correlation stats are as follows; **movement number (a)**, r = 0.59, *p* = 0.03, **horizontal activity (b)**, r = 0.65, *p* = .01, **time spent moving (c)**, r = 0.57, *p* = 0.03.

Given the relationship established between nigral DA and open-field locomotor activity (Fig. 3) as well as the role of ser31 effective stoichiometry in determining DA bioavailability (Figs. 1, 2), ser31 TH phosphorylation in SN would be predicted to correlate to inherent locomotor activity. Indeed, ser31 phosphorylation was correlated to the same locomotor activity measures as total DA or DA normalized to protein (Table 1), with the only exceptions being lowest lifetime total distance and lowest time spent moving measures. Our longitudinal characterization of locomotor activity enabled us to determine how effective stoichiometry of ser31 in SN affected locomotor capabilities. There was a significant correlation with the highest ever recorded lifetime movement number, horizontal activity, and time spent moving (Fig. 9). Therefore, nigral ser31 TH phosphorylation may regulate inherent abilities to generate locomotor activity.

## Discussion

Somatodendritic release of nigral DA has been long established [34] and its pharmacological manipulation modulates locomotor-regulating functions of the basal ganglia and aspects of locomotion [39]–[41], [59]. Furthermore, transplantation of mouse fetal midbrain cells into the SN, rather than the striatum, of 6-OHDA-lesioned adult mice may have a greater restorative capacity to locomotor function [60]. Nonetheless, it has been questioned as to whether such manipulations could somehow coincidentally modulate striatal functions. Here we show, using intact and pharmacologically-naïve rats with inherent differences in locomotor activity, that locomotor activity generation capacities in an open-field correlate to nigral, but not striatal, DA tissue content. Furthermore, the aging-related loss of DA in the SN, rather than striatum, in conjunction with a significant decline in locomotor activity in the 30-month group supports a role for nigral DA tissue content in modulating locomotor activity generation. We note that movement speed was not correlated with nigral DA tissue content and was also not significantly different between the 12 and 30 month old subjects, suggesting this component of locomotor activity is not influenced by nigral DA content. Striatal and accumbal DA content do affect motivational aspects of locomotor activity [31]–[32], [61]. However, the lack of significant DA loss in striatum or nucleus accumbens between the age groups may have accounted for why there was not a significant correlation in these regions with locomotor speed.

Well-executed aging studies that quantify striatal DA show varying amounts of tissue DA loss with age, ranging from 0 up to ∼50% [11]–[15], [17], [44], [54], [62]–[65]. However, if the widely-accepted threshold of 70–80% loss of DA neuropil is necessary for appearance of PD motor symptoms (such as bradykinesia), we must consider then how the lesser (if any in some cases) loss of striatal DA in aging still precipitates bradykinesia. In PD models, PD-like symptoms are not observed with 42–58% loss of striatal DA [10]. It is possible that striatal DA is critical for aspects of locomotor activity not related to initiating locomotor activity, such as movement speed. However, the loss of nigral DA seen in our work has also been reported in other aging studies [13], [17], [44]. Furthermore, loss of nigral TH protein of ∼40%, as seen in the 30-month group in our study (Fig. 7), is coincident with PD-like symptoms in PD models [10] and in human PD patients [42]. These observations in conjunction with our findings, which are coincident measures of both striatal and nigral DA that are associated with longitudinally-characterized locomotor activity, make a case that nigral DA has a role in modulating basal ganglia circuitry to facilitate the generation of locomotor activity.

Given that both the locomotor activity and DA and TH analyses were conducted in the same rats, a critical importance for phosphorylation at ser31 in regulating DA tissue content and a DA-dependent behavior (locomotion) was revealed. First, the differences in DA tissue content across the four regions did not correspond to total TH protein (Fig. 1, a, b). However, in conjunction with TH protein content, phosphorylation at ser31, but not ser19 or ser40, showed significant differences between striatum, SN, nucleus accumbens, and VTA that reflected the inherent differences in DA tissue content in these regions (Fig. 1, 2). The other major indication that ser31 phosphorylation affected DA bioavailability is that the significant loss of DA (along with TH protein loss) in the SN in the aged subjects was associated with a significant decrease in phosphorylation only at ser31. Together, these observations address which TH phosphorylation site regulates DA biosynthesis *in vivo* in intact neuropil: a vital question yet to be answered. However, phosphorylation at ser40 can increase DA biosynthesis [24]–[26]. It may that challenges to DA systems such as that in neurodegenerative processes could increase ser40 phosphorylation to an extent that affects DA biosynthesis capabilities [27], [28]. Still, in intact and drug-naïve DA pathways, ser31 phosphorylation and TH protein levels appear to regulate basal DA bioavailability. In the SN, these mechanisms may define the maximal locomotor capabilities inherent to our test subjects (Fig. 9). Determining whether ser31 or ser40 regulate DA biosynthesis in the TH-depleted SN in a PD model is an important next step, particularly since aging may impair compensatory processes that normally replenish DA in the face of TH loss seen in PD and PD models [65], [66].

Our assessment of locomotion took into account that the neurochemical profile attained for each test subject was only a one-time measure. In spite of definite differences in average locomotor activity shown in the test subjects, the test subjects had variable locomotor activity from trial to trial (Fig. S5). Therefore, a correlation of DA bioavailability with the 17^th^ and final trial was essential. On one hand, this correlation might be expected to be the most relevant one because the DA levels inherent in the test subject were temporally matched with the locomotor activity observed. On the other hand, indeterminate factors could also influence locomotion on a trial to trial basis, such as the subject's level of arousal, motivation for food or drink, or circadian type fluctuations that might occur within the 8 hour time window (0900 to 1700) we did our testing. Therefore, average locomotor activity results would attenuate the influence of these indeterminate effects. We found significant correlations with nigral DA content for all comparisons to movement number, horizontal activity, and time spent moving when accounting for these variables (Table 1). It is also possible that trial-by-trial variances in locomotor activity could be related to variances in DA bioavailability, which, like locomotor activity, could fluctuate around a mean value. We also point out that in the minority of observations in the 17 trials, there were some matched test subjects that, in spite of having significant differences in mean activity, had on occasion similar locomotor activity (Fig. S5). This observation suggests that other aging-related issues such as muscle function likely did not contribute to the aging-related decline of locomotor activity generation. For if aging-related muscle dysfunction did affect movement generation, an older rat would presumably never generate movement akin to that of a younger rat. The lack of correlation of central DA systems with aging-related muscle function decline has also been previously reported [67].

Aging brings an inherent risk of developing Parkinson's-like symptoms [3], [4], and their incidence increases dramatically from 15% of those at age 65 to 52% at age 85 [4]. This suggests that aging-related bradykinesia may not be universal, but the incidence certainly increases with advancing age. The neurobiological basis for the occurrence is not well understood. In our study, it appears that activity levels, which may ultimately be related to nigral ser31 and TH protein levels, at a younger age dictate the locomotor activity with advancing age (Fig. 5). There was a significant decrease in locomotor activity parameters that correlated with nigral DA (movement number, horizontal activity, and time spent moving) between 22 and 30 months of age (Fig. 4). We did not measure DA in the 22-month old rat, but there was a significant decrease in nigral DA in the 30-month rat compared to the 12-month old rat (Fig. 6). Based upon the locomotor data and the two time points at 12 and 30 months wherein DA was measured, we would predict that nigral DA loss would be less overall at 22 months than at 30 months. Taken together, these data imply that the likelihood bradykinesia occurring with aging may depend upon activity levels at a younger age, and nigral ser31 phosphorylation and TH protein levels could play a role in the inherent risk of developing aging-related Parkinsonism.

In spite of little or no loss of striatal DA tissue content in the aged test subjects, deficient DA release in aged striatum [12], [14], could impact locomotor activity. However, release capacity of nigral DA also decreases to ∼70% [14]. Therefore, both regions show diminished DA release capacity with aging. Thus the contribution of diminished DA release in either region to aging-related bradykinesia is not known. However, pharmacological blockade of nigral DA release to the extent seen in aging reduces rotorod performance [39], which suggests sufficient nigral DA bioavailability is necessary for enabling movements required to negotiate motor tasks. We show nigral DA content in the 30-month old group was ∼4.3 ng/mg protein or 6.6 ng of total nigral DA in the rostral-caudal extension and this was associated with significant reduction in locomotor activity relative to adult, 12-month old rat, which had ∼7.6 ng/mg protein or 10.3 ng total nigral DA. The significant correlation of nigral DA with locomotor activity measures (Fig. 3, Table 1) and aging-related reductions in nigral DA both make a case that nigral DA bioavailability is involved with the generation of locomotor activity.

Striatal or accumbal DA content could influence aspects of locomotion not related to its initiation and maintenance, such as movement speed [30], [61]. Notably, movement speed was not significantly different between the two age groups and did not correlate to nigral DA as did the other four parameters measured (Table 1). Thus, movement speed may be independent of the ability to initiate locomotor activity and is likely to be associated with DA signaling in striatum or nucleus accumbens, wherein DA signaling has been linked to motivational states [31], [61], [68]. We did note a trend toward significance with lifetime and second session average movement speed correlating to total striatal DA recovered. Therefore, experimental manipulation of DA in these regions beyond that produced by aging in this study (Fig. 6) could reveal a role for DA in locomotor parameters affected in PD (wherein such major loss is seen) such as movement speed, balance, gait function, and tremor at rest. It must be noted, however, that the inherent ability to generate movement, or readiness, is likely to be associated with DA bioavailability of the nigrostriatal pathway [69]. Our data suggest that nigral DA bioavailability is a significant neurobiological component in inherent abilities to initiate volitional movement.

In summary, longitudinally-characterized locomotor activity patterns of pharmacologically-naïve rats with intact nigrostriatal and mesoaccumbens pathways showed that nigral DA bioavailability, as regulated by TH protein and phosphorylation at ser31, influenced inherent capacities for the initiation and maintenance of locomotor activity. Reduced nigral, but not striatal, DA was associated with aging-related bradykinesia. We do not imply that striatal or accumbal DA has no influence upon locomotor activity, but it may be that experimental manipulation of DA levels exceeding that seen in this study (15% or less DA loss) may be necessary to determine how DA in these regions affects other locomotor parameters. We also show that in intact dopaminergic neuropil, the phosphorylation of TH at ser31, but not ser40, in addition to TH protein, governs DA bioavailability. This is not to say that ser40 phosphorylation could not influence DA biosynthesis capabilities *in vivo*, as certain pathologies or conditions might increase ser40 levels sufficiently enough to impact DA biosynthesis *in vivo* [27], [28]. However, TH protein and phosphorylation at ser31 in the SN was significantly correlated with DA tissue content and with locomotor activity levels. Therefore, studies wherein the regulation of the biosynthesis of DA or other catecholamines, like norepinephrine, is called into question *in vivo* must include analysis of ser31 phosphorylation dynamics. Future studies should address the extent to which changes in ser31 and ser40 phosphorylation, induced via experimental manipulation, affect DA biosynthesis capacity in the regions studied herein. Furthermore, modulation of DA bioavailability through inhibition or augmentation of levels in striatum and SN would determine the extent to which DA in each region modulates particular aspects of locomotor activity. In a larger realm, these results also suggest that focusing on neurotransmitter function solely at terminal fields is insufficient to understand behavior. Clearly, regulation of neurotransmitter function in somatodendritic regions must be considered as well.

## Supporting Information

Text S1 Critical supporting information for manuscript.
(0.03 MB DOC)

Figure S1 Phosphorylation-state specificity of ser31 antibody produced by 21st Century Biochemicals. Top row values are the quantity of total TH assayed to generate the phosphorylation-state specific immunoreactivity quantities for the ser31 phosphorylation site on TH (as listed in the bottom row). The relative stoichiometries of ser31 phosphorylation for the two sources used were 0.8 for the group of three standards on the left (lanes 1–3) and 0.09 for the group of three standards on the right (lanes 4–6).
(0.04 MB TIF)

Figure S2 Correlation of total TH versus total DA in the substantia nigra (SN). Spearman r = 0.656, p = 0.011. All data points represent operationally-matched results from SN dissections from the subjects used in this locomotor correlation study.
(0.01 MB TIF)

Figure S3 Correlation of movement number with other locomotor activity parameters. Data presented are means of individual locomotor tests conducted at 4 and 22 months and movement number correlated to horizontal activity (HAC) (p<0.0001; r = 0.982), total distance (cm/hr) (p<0.0001; r = 0.947), and time spent moving (p<0.0001; r = 0.937). No significant correlation of movement number to movement speed (movt velocity) (p = 0.065) was seen. Significant correlations were also observed at 12 and 30 months of age for HAC, total distance, and time spent moving.
(0.01 MB TIF)

Figure S4 Dopamine content (expressed as total harvested in the dissections) in the SN. In Figure 5 in the main text, DA content is normalized to total protein. This figure shows that total DA tissue content is reduced in the 30 month group, overall. Note there is considerable overlap in nigral DA content between the two groups, and the DA levels from each of these subjects were compared to locomotor parameters, as seen in Table 1 in main text and Table S2. Overall levels were 10.30±0.96 ng in the 12-mo group and 6.6±1.0 ng in the 30-mo group. Statistics, Unpaired Student t-test p<0.05.
(0.01 MB TIF)

Figure S5 Example of between-group differences and session-to-session variability total distance and movement number in two selected pairs of young and old test subjects. Test results are from the 12 and 30 month session. In young-1 vs. old-1, no difference in mean locomotor activity was seen in spite of the age difference, whereas in young-2 vs. old-2 a significant difference in mean locomotor activity is evident. This exception to the overall difference in locomotion with aging we report highlights that aging is not necessarily associated with bradykinesia on a case-by-case basis. Furthermore it is evident that young-1 would be at the low end of activity level for that age-group, when compared to activity seen in young-2. Also noted in the young-2 versus old-2 comparison is that in order to fully characterize locomotor activity, multiple sessions are necessary. For example, if comparing session results from the young subject that were at the low end of the locomotor activity range versus session results from the old subject at the high end of the range, as highlighted by the box-inset, little difference in locomotor activity between the subjects would be concluded.
(0.01 MB TIF)

Table S1 Habituation of locomotion.
(0.04 MB DOC)

Table S2 Mesoaccumbens pathway DA correlations (p-values).
(0.05 MB DOC)
