# Supplementary material for: Aging Reveals a Role for Nigral Tyrosine Hydroxylase ser31 Phosphorylation in Locomotor Activity Generation
Source: PLoS One. 2009 Dec 23;4(12):e8466. doi: 10.1371/journal.pone.0008466 (PMC2791868; doi:10.1371/journal.pone.0008466)
Supplement: Text S1 — Critical supporting information for manuscript. (0.03 MB DOC) [file pone.0008466.s001.doc]

**Supplemental Material**

***Aging reveals a role for nigral tyrosine hydroxylase ser31 phosphorylation in locomotor activity generation*,** M.F. Salvatore et al.

***dopaminergic and phosphorylation measures***

Protein phosphorylation is quantified by western blot using affinity-purified primary antibodies that are developed to the phosphorylation site. We have in-house our own affinity-purified ser31 phosphorylation primary (21^st^ Century Biochemicals) and ser19 and ser40 antibodies a gift from Dr. John W. Haycock. The ser31 primary antibody was validated for phosphorylation-state specificity (Fig. S1). Calibrated phosphorylation site-specific in house standards are used to quantify phosphorylation stoichiometry levels and the sample results are normalized to the TH content of each sample (Fig. 8a) to determine the phosphorylation stoichiometry. Assays for TH protein and its site-specific phosphorylation in samples are always conducted within the dynamic working range of each antibody when possible, as defined by the standard curve (Figs 7a, 8a). The total level of TH protein harvested in these samples was significantly correlated with DA tissue levels in the SN of our test subjects (Fig. S2)

***Locomotor activity correlations***

Movement number was significantly correlated with horizontal activity,total distance, and time spent moving (Fig. S3). In addition to the significant difference in DA tissue content (as normalized to total protein content (Fig. 6)) in the SN between the 12 and 30 month old groups, the total DA tissue content was also significantly different between these two groups (Fig S4). This signifies that our dissection method was consistent among the test subjects. Imprecise dissections could allow for the addition of non-dopaminergic neuropil in varying degrees, thereby introducing variance in total protein and hindering an accurate determination of DA per protein. We were interested in both the overall locomotor activity between these two populations with respect to age and also the trial-by-trial variability in individual locomotor parameters. As we are correlating regional DA levels with locomotor activity levels in each subject, the trial-by-trial variance was critical to establish as a possible confounding influence in interpreting DA correlations with locomotor activity analyses, and we addressed this aspect of our correlation of the one-time DA measure with the result obtained from the last test trial result (as seen in Table 1). We found the mean difference between the last-test trial result (the 17^th^ test) with the mean overall result (17 trials) for the test subjects was 20%, 28%, and 20% for movement number, distance traveled, and horizontal activity. In figure S5, we highlight the trial-by-trial results from the second locomotor testing period (total of seven trials) from two different pairs of 12 and 30 month old rats to stress the necessity of correlating to the very last testing trial as well as to the highest and lowest values from among the 17 trials in addition to the lifetime and second session means. This data also shows that although there are significant age group differences in locomotor activity overall (Fig. 4), there can also be similarity of mean locomotor activities between specific test subjects between the two age groups (Fig. S5 a,c) and in locomotor testing trials of the same test subjects (Fig S5 b,d). Indeterminate variables that influence locomotion such as motivational states of hunger, thirst, arousal, could influence locomotor activity on a session-to-session basis, and therefore correlation to mean locomotor activities diminishes such influences.

***Habituation of locomotion***

Animals will habituate to their environment and regarding locomotor activity, this can produce a reduction in locomotor activity as the number of trials increases until a plateau is reached [12]. We examined whether the subjects in this study exhibited these traits. If habituation occurred in the test subjects over the course of locomotor testing, this could confound interpretation of the mean locomotor results. We found that in the first session, wherein ten trials were conducted per test subject, that five of the 14 subjects exhibited significantly different movement numbers between the first five trials and the second five trials (Table S1).

In the second testing session at 12 and 30 months there were three fewer one hour trials conducted compared to the first testing session at 4 and 22 months. Therefore, we determined whether or not habituation occurred by correlating the movement number with the trial number. Only one of the 14 test subjects showed a significant correlation (negative) with decreasing movement number as the number of one hour testing sessions (or trials) increased. Therefore habituation affected a minority of the subjects and the mean lifetime locomotor activity results.
