# Supplementary material for: Aging Reveals a Role for Nigral Tyrosine Hydroxylase ser31 Phosphorylation in Locomotor Activity Generation
Source: PLoS One. 2009 Dec 23;4(12):e8466. doi: 10.1371/journal.pone.0008466 (PMC2791868; doi:10.1371/journal.pone.0008466)
Supplement: Table S1 — Habituation of locomotion. (0.04 MB DOC) [file pone.0008466.s007.doc]

**TABLE S1: Habituation of locomotion**

| **Rat ID** | **Mean ± SEM**  **session #’s 1-5** | **Mean ± SEM**  **session #’s 6-10** | ***p*** |
| --- | --- | --- | --- |
| **y-1*** | **223 ± 20** | **153 ± 17** | **0.03** |
| **o-1*** | **165 ± 6** | **118 ± 14** | **0.02** |
| **y-2** | **301 ± 22** | **258 ± 30** | **0.27** |
| **o-2** | **110 ± 30** | **82 ± 23** | **0.48** |
| **y-3** | **245 ± 29** | **183 ± 27** | **0.16** |
| **o-3** | **115 ± 10** | **118 ± 19** | **0.91** |
| **y-4** | **292 ± 30** | **202 ± 37** | **0.09** |
| **o-4** | **118 ± 16** | **95 ± 13** | **0.28** |
| **y-5** | **241 ± 32** | **146 ± 28** | **0.06** |
| **o-5*** | **147 ± 24** | **75 ± 9** | **0.02** |
| **y-6*** | **293 ± 35** | **405 ± 28** | **0.04** |
| **o-6** | **145 ± 17** | **130 ± 9** | **0.44** |
| **y-7** | **271 ± 39** | **174 ± 29** | **0.08** |
| **o-7*** | **142 ± 5** | **84 ± 6** | **0.0001** |

**Table S1.** Comparison of mean movement number between the first five and last five testing trials in the first session of young (y) and old (o) BNF F_1_ hybrid rats. Five of the 14 subjects showed a significant change in movement number between the first five and second five locomotor test trials, in one case this was an increase (y-6).
