# Supplementary material for: Aging Reveals a Role for Nigral Tyrosine Hydroxylase ser31 Phosphorylation in Locomotor Activity Generation
Source: PLoS One. 2009 Dec 23;4(12):e8466. doi: 10.1371/journal.pone.0008466 (PMC2791868; doi:10.1371/journal.pone.0008466)
Supplement: Table S2 — Mesoaccumbens pathway DA correlations (p-values). (0.05 MB DOC) [file pone.0008466.s008.doc]

**Table S2**

Correlation statistics (*p*-values derived from Spearman correlation) of locomotor activity measures to respective DA tissue content (total ng or total ng per protein) in nucleus accumbens (NAc) and ventral tegmental area (VTA) analyzed in 7 paired 12- and 30-month old rats. Coincident measures for striatum and substantia nigra are presented in Table 1 in the main text.
